# Genetic Heterogeneity, Tumor Microenvironment and Immunotherapy in Triple-Negative Breast Cancer

**DOI:** 10.3390/ijms232314937

**Published:** 2022-11-29

**Authors:** Eva Kudelova, Marek Smolar, Veronika Holubekova, Andrea Hornakova, Dana Dvorska, Vincent Lucansky, Lenka Koklesova, Erik Kudela, Peter Kubatka

**Affiliations:** 1 Clinic of Surgery and Transplant Centre, Jessenius Faculty of Medicine Martin, Comenius University in Bratislava, 03601 Martin, Slovakia; 2 Biomedical Centre, Jessenius Faculty of Medicine Martin, Comenius University in Bratislava, 03601 Martin, Slovakia; 3 Clinic of Gynecology and Obstetrics, Jessenius Faculty of Medicine Martin, Comenius University in Bratislava, 03601 Martin, Slovakia; 4 Department of Medical Biology, Jessenius Faculty of Medicine Martin, Comenius University in Bratislava, 03601 Martin, Slovakia

**Keywords:** breast cancer, triple negative breast cancer, molecular diagnostics, personalized medicine, immune system

## Abstract

Heterogeneity of triple-negative breast cancer is well known at clinical, histopathological, and molecular levels. Genomic instability and greater mutation rates, which may result in the creation of neoantigens and enhanced immunogenicity, are additional characteristics of this breast cancer type. Clinical outcome is poor due to early age of onset, high metastatic potential, and increased likelihood of distant recurrence. Consequently, efforts to elucidate molecular mechanisms of breast cancer development, progression, and metastatic spread have been initiated to improve treatment options and improve outcomes for these patients. The extremely complex and heterogeneous tumor immune microenvironment is made up of several cell types and commonly possesses disorganized gene expression. Altered signaling pathways are mainly associated with mutated genes including p53, *PIK3CA*, and *MAPK*, and which are positively correlated with genes regulating immune response. Of note, particular immunity-associated genes could be used in prognostic indexes to assess the most effective management. Recent findings highlight the fact that long non-coding RNAs also play an important role in shaping tumor microenvironment formation, and can mediate tumor immune evasion. Identification of molecular signatures, through the use of multi-omics approaches, and effector pathways that drive early stages of the carcinogenic process are important steps in developing new strategies for targeted cancer treatment and prevention. Advances in immunotherapy by remodeling the host immune system to eradicate tumor cells have great promise to lead to novel therapeutic strategies. Current research is focused on combining immune checkpoint inhibition with chemotherapy, *PARP* inhibitors, cancer vaccines, or natural killer cell therapy. Targeted therapies may improve therapeutic response, eliminate therapeutic resistance, and improve overall patient survival. In the future, these evolving advancements should be implemented for personalized medicine and state-of-art management of cancer patients.

## 1. Introduction

Breast cancer is a leading cause of cancer death in women worldwide [1]. Historically, breast cancer classification has been based on morphologic findings; however, resent advances have taken into account molecular biomarkers, genetic alterations, and clinical features [2]. Triple negative breast cancer (*TNBC*) represents 15–20% of all breast cancers and is characterized by the lack of estrogen receptor (*ER*) and progesterone receptor (*PR*) expression and the absence of *HER2* overexpression or amplification [3]. *TNBC* is considered the most aggressive breast cancer subtype with the least favorable clinical outcomes [4].

A disease recurrence will occur in approximately 50% of *TNBC* patients who underwent radical surgery without metastasis [5]. When compared to other breast cancer subtypes, *TNBC* displays an increased pathological complete response (*pCR*) rates (7% vs. 22–45, respectively) [6]; however, *TNBC* patients display higher mortality. Specifically, 37% mortality within the first 5 years of diagnosis [7] and worse overall survival [8] when a complete response is not achieved. There are no approved targeted treatments for *TNBC*, in contrast to other breast cancer subtypes, which leverage therapeutic targets such as ER or HER and systemic chemotherapy continues to be the gold standard of care. Chemotherapy responses are typically transient, making this disease subtype difficult to treat clinically.

The inherent heterogeneity of *TNBC* is well-known at clinical, histopathological, and molecular levels [9]. Genomic instability and greater mutation rates, which may result in the creation of neoantigens and enhanced immunogenicity, are additional characteristics of *TNBC* [10,11]. *TNBC* is the most immunogenic breast cancer subtype, with higher PD-L1 expression and more tumor infiltrating lymphocytes (*TILs*) [12]. The complex and heterogeneous tumor immune microenvironment (TIME) in *TNBC* is composed of several cell types and exhibits non-uniform gene expression [13]. It also bears consideration that the fast growth rates generally seen in *TNBC* results in hypoxia and necrosis, which reprograms the TIME gene expression landscape and significantly alters immune cell survival, tumor detection, and anti-tumor activity [14,15].

## 2. Molecular Classification of *TNBC*

*TNBC* demonstrates genomic heterogeneity, as there is substantial variation in mutational burden, copy number alterations, and genomic rearrangements across *TNBC* patients [16]. Previous studies have defined subtypes of *TNBC* based on gene expression [17,18,19], and such efforts achieved significant progress in classifying *TNBC*. By analyzing publicly available expression data for messenger RNA (mRNA), Lehmann et al. [20] acknowledged *TNBC*, and classified *TNBC* into six subtypes (i.e., *TNBC* type-6 classification) with the remainder classified as an unstable type (*UNS/UNC*) [21,22,23]. Further, the Lehmann et al. work has revealed that an abundance of either infiltrating lymphocytes or tumor-associated stromal cells within the sample was the primary determinant specifying an either immunomodulatory (*IM*) or mesenchymal stem-like (*MSL*) subtype, resulting in a consensus of four intrinsically defined *TNBC* subtypes refined as basal-like 1 and 2, (*BL1*, *BL2*), mesenchymal (*M*), and luminal androgen receptor (*LAR*) (*TNBC* type-4 classification) [19].

In 2015, Burstein et al. [18] suggested dividing *TNBC* into two major groups based on quantitative DNA expression which were further categorized into four subtypes based on identified potential targets [10]. These targets included the *LAR* group and a group consisting of three other subtypes with similar gene expression patterns. These other subtypes are the mesenchymal subtype (*MES*), the basal-like immunosuppressed (*BLIS*) subtype which expresses the immunosuppressive molecule V-Set Domain Containing T-cell activation inhibitor 1 (*VTCN1*), and the basal-like immune-activated (*BLIA*) subtype which exhibits activation of the signal transducer and activator of transcription (STAT) [24].

More recently, Liu et al. [21] combined mRNA and long non-coding RNA (lncRNA) expression profiles in *TNBC* to create the Fudan University classification (FUSCC) system. This classification system has four distinct subtypes of *TNBC*, specifically, *IM*, *LAR*, *MES*, and *BLIS*.

As shown in Table 1, all available classification systems do contain a subtype characterized by distinct gene expression associated with the immune system. To address this issue, we need to explore more deeply the tumor immune drivers associated with different subtypes and stages of *TNBC* to provide immunotherapy more effectively to these patients. The study of Hu et al. identified three *TNBC* immune subtypes, termed IS 1–3 (with subclassifications including IS3A and IS3B) and observed significant differences in prognosis, sensitivity to immunotherapy and chemotherapy, gene mutation, and immune infiltration. Their findings represent a clinical foundation for the classification and additional immunotherapy of *TNBC* based on seven functional modules of immune-related genes [25].

## 3. Genomic and Transcriptomic Landscape of *TNBC* with Focus on the Immune System

Identification of genes likely responsible for *TNBC* is possible by using emerging technologies such as DNA copy number assays, mRNA and protein arrays, DNA methylation profiling; exon expression, and RNA or microRNA profiling using next generation sequencing (NGS). Knowledge of the molecular background of *TNBC* may impact on characterization and diagnosis of *TNBC* and inform the search for personalized therapies. *TNBC* exhibits a high proportion of mutations which result in an unstable genome and may potentially stimulate an immune response [12].

### 3.1. Transcriptional Landscape of TNBC

Gene expression profiling of *TNBC* tissues revealed that deficiencies or mutations in *BRCA1* or *BRCA2* genes were present in up to 20% of women [26] and *BRCA1* mutation is significantly associated with positive CK5/6 expression [27]. Another study found an overexpression of Poly(ADP-ribose) polymerase 1 (*PARP-1*) enzyme in tissue of *TNBC* patients by RNA microarray, and this was validated by immunohistochemistry [28]. *PARP-1* expression has also been detected in circulating tumor cells in blood plasma of breast cancer patients and could be used in prediction of therapy selection and patient outcome [29]. A separate study found that Basic Helix–Loop–Helix Family Member E41 (*BHLHE41* or *SHARP1*) is a key regulator of the invasive and metastatic phenotype of the most aggressive forms of *TNBC*. Levels of *BHLHE41* expression inversely correlate with hypoxia-inducible factors HIF1A and HIF2A and cause proteasome degradation of HIFs [30].

In African breast cancer patients, a higher expression of aldehyde dehydrogenase 1 (*ALD1*) was found to be associated with a higher mitotic rate, high histologic grade, and ER/PR negativity, confirming its more aggressive phenotype [31]. Upregulation of the polymorphic and/or ancestry-specific gene expression was found in PIM3, ZBTB22, and PPP2R4 genes in *TNBC* tissues of African ancestry. It is thus necessary to investigate populations of various origins to evaluate a molecular diversity of *TNBC* [32].

### 3.2. Genomic Profile of TNBC Tissue

The most frequently occurring somatic mutation in 104 *TNBC* cases was found in tumor protein p53 (*TP53*) gene where 43% of non-basal *TNBC* and 62% of basal *TNBC* cases carried at least one mutated allele. Other frequently mutated gene is the Phosphatidylinositol-4,5-Bisphosphate 3-Kinase Catalytic Subunit Alpha (*PIK3CA*) gene at 10.2%, Usherin (*USH2A*) gene at 9.2%, Myosin 3A (*MYO3A*) gene at 9.2%, and Phosphatase and Tension Homolog (*PTEN*) and retinoblastoma 1 (*RB1*) gene at 7.7%. Other frequently mutated genes were *ATM*, *UBR5* (*EDD1*), *COL6A3*, *GH1*, synuclein genes (*SYNE1/2*, *BRCA2*, *BRAF*, *NRAS*, *ERBB2*, and *ERBB3*, as well as somatic mutation *BRAF* V600E, and significant *EGFR* amplification [33]. Similarly, in a Chinese population, *TP53* mutations were found in 74% of *TNBC* tumors, followed by mutations on *PIK3CA* (18%), *KMT2C* (7%), and *PTEN* (6%) genes [34]. Another study also confirmed a positive correlation of *TP53* mutations in 62% of *TNBC* [35]. Other frequently mutated genes were *PIK3CA* in 17% of cases, *APC* in 3%, *RET* in 3%, *SMAD4* in 3%, and in 1% of cases mutations in *AKT1*, *ATM*, *BRAF*, *FGFR1*, *HRAS*, *JAK3*, *MET*, *SRC*, *PTEN*, and *STK11* [35] were observed. Other DNA microarray analysis of *TNBC* tissues identified 104 upregulated genes, such as NIMA-related kinase 2 (*NEK2*), PDZ binding kinase (*PBK*), denticleless homolog (Drosophila) (*DTL*), maternal leucine zipper kinase (*MELK*), and kinesin family member C (*KIF2C*). These are functionally inactivated in cell–matrix or cell–cell interactions, and are responsible for nuclear division, chromosome segregation, kinetochore, microtubule organization, and epithelial-to-mesenchymal transition (*EMT*) [36]. The clonal evolution of primary heterogenous *TNBC* appears most frequently from drivers such as *TP53* and *PIK3CA*/*PTEN*, and at a lower frequency, in ECM signaling genes and cell motility/shape. We suggest that a different phase of molecular progression may be found at the time of primary *TNBC* diagnosis, and that subsequent approaches for ‘low clonality’ and ‘high clonality’ primary tumors should be selected [33].

A signaling pathways altered in *TNBC* are mainly associated with mutated genes outlined above, including p53 signaling pathway (*TP53* mutations), Mismatch DNA repair (*MMR*) pathway, *BRCA* 1/2 mutations, PIK3/AKT mutations, and MAP3K1 mutation and signaling dysregulation, as well as estrogen pathways which typically exhibit lower activity in *TNBC* due to lack of ER expression [37].

The most frequently altered genes in *TNBC* are displayed in Table 2.

### 3.3. Genomic Alterations and Their Impact on Immune Surveillance

Tumor cells carrying various mutations may be either eliminated by the immune system as a part of cancer immunosurveillance, tolerated by the immune system as a latent period of immune-mediated equilibrium after incomplete destruction of tumor, and/or escape the immunological restraints of the equilibrium and tumor growth [65]. A study revealed more somatic mutations and higher expression levels of HLA genes, and possibly more HLA-binding peptides in *TNBC* [37]. Moreover, this finding indicates a stronger immunogenicity in *TNBC*. *TNBC* has also been described via the expression of immunogenic proteins such as cancer-testis (*CT*) antigens that are normally expressed in the human germ line. The increased levels of *CT* gene expression were found in high-grade and *TP53*-mutated *TNBC*. *TNBC* tissue was found with increased expression of cytokine receptor genes, such as *CCR1*, *CCR3*, *CCR5*, *CCR7*, *CCR8*, and *CCR9* [37].

The host immune response to tumors and cancer treatment is influenced by inflammation, as well as tumor development is often associated with an inflammatory response [66]. Gene expression analysis uncovered that 14 proinflammatory genes (*CCL5*, *CD19*, *CD88*, *CXCL10*, *CXCL13*, *CXCL9*, *GNLY*, *GZMB*, *IFNG*, *IGKC*, *IL12B*, *IRF1*, *PRF1*, and *TBX21*) were significantly overexpressed in *TNBC* when compared to other breast cancer subtypes or normal breast tissue. Authors concluded that *TNBC* tissue displays stronger immune activities and inflammatory response [37]. The *IM* subtype of breast cancer is associated with elevated expression of immune-related genes, as *BIRC3*, *BTN3A1*, *CSF2RB*, *GIMAP7*, *GZMB*, *HCLS1*, *LCP2*, and SELL, and is positively correlated with expression of *PD-L1*, *PD-1*, and *CTLA4*. The overexpression of eight of the afore mentioned genes in the *IM* subtype of *TNBC* was associated with favor survival of related *TNBC* patients [67]. *TNBC* tissue has also significantly higher expression levels of Signal transducer and activator of transcription 1 (*STAT1*). *TP53*-mutated *TNBC* also showed higher expression of genes associated with a low-grade inflammatory reaction (*AIM2*, *CD14*, *CD276*, *HMOX1*, *LGMN*, *MX2*, *MMP7*, and *TLR2*) known as parainflammation. This feature may contribute to the invasiveness of *TNBC* [37].

Genes that regulate immune response correlated positively with p53, *PIK3/AKT*, and *MAPK* pathways, and negatively with *MMR* and estrogen pathways. *CT* gene sets had a negative correlation with *MMR*, *PIK3/AKT*, and *MAPK* pathways. It was also suggested that increased expression levels of genes linked to immune function are likely associated with better survival of *TNBC* patient [37].

Based on significant amounts of data obtained from high-throughput sequencing and technological advances in bioinformatics and biological computing algorithms, it is possible to perform a more complex molecular profiling of tumors and to identify previously unrecognized molecular biomarkers. Weighted gene co-expression network analysis (WGCNA) showed a significant association of *AIM2*, *CCL5*, and *CCL25* genes with the overall survival of *TNBC* patients. Based on these findings, Wang et al. constructed the Immune-Related Gene Prognostic Index (*IRGPI*) involving only genes *CCL5* and *CCL25* (*IRGPI* = expression level of *CCL25* × (−1.1233) + expression level of *CCL5* × (−0.0033)). The *IRGPI*-high subgroup was correlated with the abundance of M0 and M2 macrophages, whereas the *IRPGI*-low subgroup was characterized by the dominant presence of cytotoxic *CD8* T cells, *CD4* T cells, and M1 macrophages. These results suggested that the *IRGPI*-low subgroup had a favorable immune microenvironment when compared to the *IRGPI*-high subgroup. Moreover, *IRPGI* was shown as an independent prognostic factor of overall survival [68]. Nevertheless, these analyses require further verification to confirm associations between cancer genotype and phenotype.

## 4. Novel Findings in Epigenetics, Genetics, Non-Coding RNAs, and Biomarkers in *TNBC*

Recent research highlights the fact that long non-coding RNAs also play an important role in a tumor microenvironment formation and can mediate tumor immune evasion [69,70]. Furthermore, their levels can play an important role in *TNBC* prognosis. In silico analyses of four lncRNA datasets of *TNBC* from The Cancer Genome Atlas (*TCGA*) and gene Expression Omnibus (GEO) databases [71] demonstrated that up to 61 long non-coding RNAs (lncRNA) can be considered as immune-related in function. In addition, four lncRNA (RP11-890B15.3, RP11-1024P17.1, MFI2-AS1, and RP11-180N14.1) had high prognostic value in *TNBC*. Based on their expression levels, it was possible to create a prognostic risk model that divided *TNBC* patients into high and low-risk groups. Based on gene set variation analysis, it was determined that patients from the high-risk group had markedly enriched response to tumor cells, humoral immune response, and high levels of various infiltrating lymphocyte types. Finally, four lncRNAs were shown to have a significant effect on the development of several cancer types by various mechanisms, predominantly by sponging regulatory miRNAs [72,73,74,75].

MicroRNAs (miRNAs) can also be used as a marker of *TNBC*. Several studies have demonstrated that miRNA levels are significantly abnormal in breast cancer tissues, including *TNBC*, and that their levels are altered in liquid biopsy samples from breast cancer patients as well [76,77,78,79]. It is highly likely that change in miRNA expression and activity can be associated with abnormalities in the immune response in *TNBC* [80]. miRNAs are considered important regulators of both innate and adaptive immune response, and their activity has significant effects on the development and differentiation of various immune cells [81]. Finally, miRNAs have the ability to regulate immune and cancer cell interactions within the TME, thus controlling both the pro- and anti-tumor immune responses [82].

MiR-10b and miR-20a have been noted to regulate the expression of ligands that bind to *NKG2D* receptors on the surface of natural killers (*NK*) cells, reducing the final cytotoxicity of these cells [83,84]. miR-19a plays an important role in the polarization of tumor-associated macrophages to the M1 phenotype in breast cancer, resulting in a reduction in the metastatic potential of these tumors [85]. Lastly, miR-126 regulates expression of *SDF-1A* and *CCL1* genes which has a significant impact in reshaping tumor-infiltrating lymphocytes [86].

Based on available data and in silico modelling, Pina-Sanchez et al. [80] demonstrated that altered activity of some miRNAs in *TNBC* patients significantly interact with immune response checkpoint genes such as *PD-1*, *PD-L1*, and *CTLA4*, and two circulating miRNAs (miR-195 and miR-155) interacted most significantly with these genes. Other miRNAs, in particular, miR-10b, miR-19a, miR-20a, miR-126, and miR-155 [87], also found to be abnormally expressed in plasma samples of breast cancer patients. Based on their expression, it was even possible to differentiate the cohort of patients with early versus metastatic breast cancer and expression levels could also be associated with disease-free survival (DFS) and overall survival (OS). Notably, low expression of miR-155 in the subgroup of patients with *TNBC* was predictive for DFS and a combined model of miR-126 and miR-155 had sufficient predictive value in discriminating relapsed from non-relapsed patients with *TNBC*. Such results of miRNA levels from liquid biopsy samples can therefore serve as independent indicators to guide neoadjuvant treatment regimens.

In *TNBC*, miRNAs have an important effect on the regulation of PD-L1 expression. Based on a systematic search of eight public databases [88], it was found that nine miRNAs (miR-424-5p, miR-138-5p, miR-570-3p, miR-200c-3p, miR-383-5p, miR-34a-5p, miR-3609, miR- 195-5p, and miR-497-5p) have the ability to inhibit PD-L1 activity in *TNBC* tissues. Their activity has a further effect on the transformation of tumor microenvironment and the regulation of several oncogenic signaling pathways, reducing the oncogenic and metastatic potential of these cells. miRNA expression profile obtained from two public datasets (*TCGA* and *GEO*) was also analyzed [89]. miRNAs with similar expression were further analyzed using logistic regression and Gaussian mixture analyses. As a result, eight miRNAs possessed the highest informative value in the prognosis of *TNBC* relapse, namely, miR-139-5p, miR-10b-5p, miR-486-5p, miR-455-3p, miR-107, miR-146b-5p, miR-324-5p, and miR-20a-5p. While the authors did not directly demonstrate association of these miRNAs with tumor microenvironment and immune system, this possibility was suggested based on the fact that these miRNAs are involved in immune response pathways [89]. The top 10 gene onthology (GO) pathways included lymphocyte activation, cell–cell adhesion, and localization to external leaflet of the plasma membrane.

In addition to miRNAs and lncRNAs, circulating tumor DNA (ctDNA) and circulating tumor cells (*CTC*) can also be used as markers of *TNBC*. Since a large proportion of patients with *TNBC* are treated with neoadjuvant therapy, both ctDNA and CTC levels were monitored following treatment [90]. The results of next generation sequencing analysis from 196 patients with early *TNBC* were used for this study. These investigators found that increased levels of ctDNA released into the blood stream from primary tumors was directly proportional a decrease in distant disease-free survival (*DDFS*). Specifically, 24 months after treatment, only 56% of ctDNA positive patients achieved *DDFS*, whereas 81% of ctDNA-negative patients achieved *DDFS*. Moreover, ctDNA positivity was also associated with reduced disease-free survival (*DSF*) and OS. A model that used a combination of ctDNA and CTC was significantly less favorable to positive patients. Both ctDNA and *CTC*-positive patients demonstrated *DDFS* after 24 months (52%) compared to negative patients (89%). In summary, ctDNA positivity had a greater impact compared to CTC positivity alone [90].

In addition to more conventional intracellular markers, tumor-microenvironment markers, such as the level of hypoxia or pH, can be utilized for *TNBC* diagnosis and predication of model of tumor growth and spread. The specific formation of the tumor microenvironment is associated with abnormalities in expression of various immune-related genes [91,92,93,94]. Hypoxia, which is typical of fast-growing breast tumors, is linked to metabolic reprogramming, stem cell signatures, angiogenesis, extracellular matrix organization, and cancer cell metastasis [95,96,97]. Zheng et al. [98] analyzed the relationship between hypoxia and immune response in *TNBC* patients. After performing analyses under in silico conditions, these investigators collected data from available datasets and subsequently validated these results with laboratory experiments on tumor tissues. Both approaches demonstrated that the expression profile of hypoxia-related genes was negatively associated with immune response and the activity of cytotoxic lymphocytes. Based on a hypoxia-immune gene signature, it was possible to distinguish *TNBC* patients into two groups, specifically, hypoxia high/immunity low and hypoxia low/immunity high subgroups. Patients in the latter group had better OS and the most significant difference in expression between these two groups was in genes *CA-IX* and *PD-L1*. Expression of *PD-L1* was statistically significantly lower in the former group while *CA-IX* expression was significantly higher in the latter.

## 5. Tumor Microenvironment, Tumor Infiltrating Lymphocytes (*TILs*) and Their Role in Disease Prognosis in *TNBC*

Breast cancer development and progression are enabled and mediated not only by the proliferation of neoplastic cells, but also by numerous interactions with heterogeneous segments that create the surrounding tumor microenvironment. Such important factors are tumor-infiltrating lymphocytes, complex myeloid cells, lipid-associated macrophages, cancer-associated fibroblasts (*CAFs*), and other molecules that promote the growth and migration of tumor cells [99]. Tumor-infiltrating lymphocytes (*TIL*) are considered an important prognostic factor in various types of oncological diseases [100,101,102,103,104] including triple-negative breast cancer (*TNBC*) [105,106]. In general, tumor-infiltrating lymphocytes are strong indicators of tumor immunogenicity [107]; however, as a heterogeneous population, *TILs* are associated both with positive and negative outcomes. Indeed, the immune status of tumors in situ and the presence of a particular subtype of lymphocytes are significant factors in predicting patient survival [108,109]. Certain types of immune cells, such as NK cells, cytotoxic T cells, and B cells, are able to suppress cancer growth and, moreover, higher amounts of immune cells are associated with a better prognosis. In contrast, other types of immune cells, including *FOXP3+* Tregs, are known to facilitate and promote carcinogenesis and tumor growth [109] although Matsumoto et al. have questioned this generally accepted opinion concerning *FOXP3+* cells [110].

In patients with *TNBC*, the *pCR* rate was significantly higher in those with tumors showing high *TIL* scores than those with low *TIL* scores. A large volume of lymphocytic infiltrate was a significant indicator of longer distant metastasis-free survival [111]. In several studies, changes in *TIL* score or the percentage in a specific subset of T-cells were shown to be correlate *pCR* to neoadjuvant chemotherapy in breast cancer. For example, an immunologic profile combining the absence of immunosuppressive *FOXP3* cells, and the presence of a high number of *CD8* T cells and cytotoxic cells, was associated with a better prognosis [111,112]. It was also demonstrated that *TIL* levels were lower in pre-chemotherapy when compared to post-chemotherapy samples and this supports the hypothesis that chemotherapy could induce an antitumor immune response [113]. It has also been observed that, when using neoadjuvant chemotherapy (*NACT*), increased *TILs* were associated with higher *pCR*, regardless of the type and duration of *NACT* therapy [114]. Patients with high-*TIL* residual disease experienced a significantly better metastases-free survival (*MFS*) when compared with patients in the low-*TIL* group [113].

*TILs* are reproducible biomarkers, and multiple studies have confirmed their prognostic value in *TNBC* (Table 3 and Table 4) [114]. Immune response mediated by *TILs* is an important factor that has to be taken into consideration in treatment management; however, the presence or absence of particular subtypes and their ratio in cell population significantly influences the outcome.

## 6. Current Immunotherapeutic Strategies of *TNBC*

Advances in immunotherapy, through remodeling the host immune system to eradicate tumor cells, could lead to the introduction of novel therapeutic strategies for *TNBC* [126,127]. Current research on this subject is focused on combining immune checkpoint inhibition with chemotherapy, *PARP* inhibitors, cancer vaccines, or *NK* cell therapy [128]. Furthermore, nutraceuticals are commonly used in the prevention and treatment of *TNBC* because plant-based food rich in phytochemicals can effectively modulate immune checkpoint-related signaling molecules such as *PD-1*/*PD-L1* [129,130]. Moreover, specifically designed nanoparticles with ligands on their surface can facilitate drug delivery by targeting to cancer cells to, for example, modulate immune responses. Additionally, nanodrug delivery systems can represent an important way to overcome multidrug resistance. However, their use is complicated due to several limitations, including poor oral bioavailability, inadequate pharmacodynamic properties, or non-selectivity [129,131].

In various cancer types, immunotherapeutic approaches include the blockade of immune checkpoints through inhibitors, adoptive cell transfer-based therapy, activation of cytotoxic T lymphocytes, and modulation of the tumor microenvironment to facilitate *CTL* activity [132]. The most well-described immune checkpoint inhibitors (ICIs) are the cytotoxic T lymphocyte-associated molecule-4 (*CTLA-4*), programmed cell death receptor-1 (*PD-1*), and programmed cell death ligand-1 (*PD-L1*) [133]. *PD-L1*, characterized by high mutagenic activity, is overexpressed in 20% of *TNBC* patients, making *PD-L1* a potential therapeutic target [134]. For example, combined analyses of *PD-L1* and tumor-infiltrating lymphocytes (*TILs*) can inform *TNBC* patient prognosis [135]. It is important to consider the immune subtype of *TNBC*, either neutrophil-enriched (*NES*) or macrophage-enriched subtypes (*MES*), especially due to differing responses to immune checkpoint blockade [136].

Certain drugs that can interrupt immune checkpoint mechanisms, including anti-*CTLA-4*, anti-*PD-1*, and anti-*PD-L1*, could mediate durable cancer regression [132]. To date, the US Food and Drug Administration has approved several monoclonal antibodies that function by blocking immune checkpoint activity. These include the anti-*CTLA*-4 antibody ipilimumab, anti-*PD1* antibodies pembrolizumab, (FDA approved drug for high-risk, early-stage *TNBC*), nivolumab, cemiplimab, and the anti-*PD-L1* antibodies atezolizumab (first FDA-approved immunotherapy drug for locally advanced or metastatic *TNBC*), avelumab, and durvalumab [137].

Several immune checkpoint inhibitors (*ICIs*) are effective in treating melanoma, non-small cell lung carcinoma, and renal cell carcinoma; however, in certain studies, as described below, some of these therapeutics have potential to treat highly metastatic *TNBC* as well. The efficacy and relative safety of a monotherapy with the anti-PD-L1 antibody atezolizumab were demonstrated in a phase I trial in metastatic *TNBC* patients [138,139]. Monotherapy using the anti-*PD-1* antibody pembrolizumab, demonstrated safety, durable antitumor activity, and was well-tolerated in patients with previously treated metastatic *TNBC* [140]. When compared with ICI monotherapy, the combination therapy of *ICIs* coupled with other therapeutic approaches appears to be more beneficial for patients with early or advanced *TNBC*. For example, atezolizumab or pembrolizumab in combination with chemotherapy were well-tolerated in phase III clinical trials in metastatic PD-L1-positive *TNBC* patients [141]. Moreover, the combination of atezolizumab and nab-paclitaxel is considered as a standard treatment for advanced PD-L1-positive *TNBC* and, moreover, appears to be safe and clinically active in a phase 1b study of stage IV or locally recurrent *TNBC* [141,142].

To date, single-agent therapy with the anti *CTLA-4* antibody ipilimumab in *TNBC* patients has received limited focus in the literature. However, one case report described a patient with metastatic *TNBC* treated with the combination of ipilimumab plus nivolumab (*PD-1* antibody), along with *IL-2*, and locoregional and whole-body hyperthermia. This combination resulted in complete clinical remission [143] in this patient. Accounts of clinical trials using nivolumab, cemiplimab, and/or durvalumab in *TNBC* patients remain absent in the literature.

Importantly, *TNBC* requires DNA damage response for survival. Alterations of DNA damage responses pathways make *TNBC* particularly sensitive to specific inhibitors such as high sensitivity of *TNBC* tumors containing *BRCA1/2* mutations to poly-ADP-ribose polymerase (*PARP*) inhibitors [144]. Therefore, *PARP* inhibitors such as veliparib, niraparib, rucaparib, olaparib, and talazoparib, commonly are combined with ICIs, especially due to their synergism with immunotherapy [145,146].

Another immunotherapeutic approach in *TNBC* is the exploitation and recruitment of NK cells. This approach promises to harness innate rather than adaptive immunity, and could potentially circumvent the undesirable effects of ICIs [147]. Chumsri et al. [148] concluded that intratumoral *CD56*-positive NK cells are linked with improved outcome in *TNBC*. In a related study, the antigen-specific antibody favored tumor and metastasis tissue infiltration by cytokine-induced NK cells and led to an increase in the *CD16a^+^* subpopulation. These data point to a nonspecific NK cell population that can be recruited as tumor-specific effectors with clinical-grade antibodies [149].

Cell surface protein-derived multi-epitope vaccine-mediated targeting of *TNBC* cells could represent an improved clinical tool to combat this type of cancer. Antigenic epitopes (selected or fused) of the cytotoxic and helper T-lymphocytes could serve for the construction of the multi-epitope vaccine (MEV). In silico models showed that specifically designed vaccine has capability to evoke immune response that can be applied to target *TNBC* alone or in combination with other therapies. Toward that end, comprehensive experimental research is warranted to evaluate the efficacy of such vaccines [150].

Several other drug combinations, as potential immunotherapeutic strategies for *TNBC*, are currently undergoing clinical studies. Table 5 summarizes the completed clinical trials of immunomodulators in immunotherapeutic strategies tested as *TNBC* treatment approaches; however, some completed clinical trials (NCT05609903, NCT03101280, NCT03292172, NCT03800836, NCT03256344, NCT03289819, NCT01676753, or NCT02900664) have not provided results or publication yet. Although use of *ICIs* can result in clinical remission in many metastatic cancer types, efficacy of *ICIs* in breast cancer, especially *TNBC*, is low [151]. Therefore, it is important to develop novel strategies for improvement of anticancer immune responses and extend survival in patients with metastatic disease such as *TNBC*. For better therapeutic response, *ICIs* should be combined with chemotherapy, targeted therapies, or other novel immunotherapies. According to available clinical evidence, ICIs appear to be well-tolerated and relatively safe; however, these findings are still in the early stages and further analysis is needed. In conclusion, combining *ICIs* with chemotherapy, *PARP* inhibitors, *NK* cell therapy, or cancer vaccines demonstrates clear potential to improve clinical management of *TNBC*.

## 7. Conclusions

*TNBC* is a delicate issue for oncologists and surgeons since this form of breast disease has generally poor prognosis and complicated treatment options. Available evidence provides a solid foundation for *TNBC*’s complex molecular portrait. The immune component of tumors has already been intensively studied. This point, ably illustrated in the work of Hanahan and Weinberg, suggests that the ability of tumors to evade attack and elimination by the immune system through a process termed immunoediting, is a hallmark of cancer [161]. Integrative models and careful investigation of immune regulation in *TNBC* are therefore promising grounds on which to fight this group of malignant breast tumors.

Due to the aggressive nature of *TNBC*, there is a critical need for prevention-based approaches. Identification of molecular signatures and effector pathways driving early stages of cancer using multi-omics approaches represents an important step toward developing new strategies for targeted prevention. A more advanced understanding of the immune system allows us to focus our research on more emerging tools, including vaccine-mediated targeting of *TNBC* cells.

Targeting the DNA repair and response mechanisms, p53, and cell proliferation mechanisms are some of the therapeutic targets for *TNBC* management. New therapy targets found in recent molecular characterization of *TNBCs* include tyrosine and non-tyrosine kinases, *PARP1*, *AR*, immune-checkpoints, and epigenetic proteins [162]. Great effort is being made to find suitable therapies for *TNBC* patients via the targeting of a specific molecular features of this tumor type.

*TNBC* is a highly heterogenous disease and individual driver genes and mechanisms should be identified to specify the therapeutic target. Recent improvements in whole genome sequencing allow us to identify the most frequently mutated genes, the genetic profile most likely responsible for the cancer development, as well as those linked to metastatic *TNBC*. Emerging targeted therapies may offer improvements to therapeutic response, eliminate resistance to therapeutics, and improve overall patient survival. In the future, such personalized medicine ideas should be implemented for use in state-of-art management of *TNBC* patients.

## Figures and Tables

**Table 1:** *TNBC* classification systems based on different signaling pathways [18,20,22,23].

**Lehman’s Subtypes**	
*BL1*	↑ Ki67 ↑ cell cycle and DNA damage response gene expression
*BL2*	↑ growth factor signaling ↑ myoepithelial markers
*M*	deregulation of *EGFR*, *MAPK*, and *PI3K* signaling
*MSL*	deregulation of *EGFR*, calcium signaling, *MAPK* ↓ genes associated with cellular proliferation ↑ genes related to mesenchymal stem cells
*IM*	↑ *IFNα* and *IFNγ* signaling ↑ cytotoxic T-lymphocyte-associated protein 4
*LAR*	↑ androgen receptor (*AR*) expression
**Burstein’s Subtypes**	
*MES*	↑ cell cycle, mismatch repair and DNA damage networks ↑ hereditary breast cancer signaling pathways
*BLIA*	↑ genes controlling B cell, T cell and natural killer cell functions
*BLIS*	↓ B cell, T cell, and natural killer cell immune-regulating pathways ↓ cytokine pathways
*LAR*	↑ *AR*, *ER*, prolactin, and *ErbB4* signaling
**FUSCC Subtypes**	
*MES*	↑ *ECM*-receptor interaction, focal adhesion, *TGF*-beta signaling ↑ *ABC* transporter ↑ Adipocytokine signaling
*IM*	↑ Cytokine–cytokine receptor interaction ↑ T and B cell receptor signaling ↑ Chemokine signaling, *NF*-kappa-B signaling
*BLIS*	↑ Mitotic cell cycle, mitotic prometaphase, M phase of mitotic cell cycle ↑ DNA replication, DNA repair ↓ Innate immune response ↓ T cell receptor signaling
*LAR*	↑ Steroid hormone biosynthesis ↑ Porphyrin and chlorophyll metabolism ↑ PPAR signaling pathway ↑ Androgen and estrogen metabolism

(*BL*, basal-like; *M*, mesenchymal; *MSL*, mesenchymal stem-like; *IM*, immunomodulatory; *LAR*, luminal androgen receptor; *MES*, mesenchymal; *BLIA*, basal-like immune-activated; *BLIS*, basal-like immunosuppressed; *ECM*, extracellular matrix; *PPAR*, peroxisome proliferator-activated receptor; *TGF*, transforming growth factor; *IFN*, interferone; *EFGR*, epidermal growth factor receptor; *MAPK*, mitogen-activated protein kinase; *PI3K*, phosphatidylinositol 3-kinase).

**Table 2:** Genes that are differentially expressed in 10% or more of *TNBC* cases.

Gene	Function	Type of Change	Prognostic Significance	Predictive Significance	Mutation Frequency	References
*TP53*	Regulate cell cycle progression, DNA repair, cellular senescence, apoptosis	Inactivating mutation	Poor prognostic factor	*Tp53* mutation status could be a useful biomarker in stratifying BC patients responsive to immunotherapy	60–88%	[38,39,40,41]
*PIK3CA*	Regulate cell proliferation differentiation, survival	Activating mutation	Negative prognostic factor	Predictive biomarker for response to chemotherapy	10.2–30.8%	[27,28,29,42,43,44,45]
*AKT1*	Survival, cell growth, cell cycle regulation, metabolism	Activating mutation	Poor prognostic factor		1–7.7%	[29,42,45,46,47]
*PTEN*	Cell proliferation, migration, invasion	Inactivating mutation	Poor prognostic factor		1–11.2%	[27,28,29,48,49]
*VEGFA*	Angiogenesis	Mutation, overexpression			30–60%	[50,51,52]
*BRCA1/2*	DNA damage repair, cell cycle control, apoptosis	Inactivating mutation	Poor prognostic factor	Potential predictor for response to *PARP* inhibitors	10–20%	[52,53,54,55,56,57]
*ATM*	Cell cycle control, apoptosis, oxidative stress	Mutation	Poor prognostic factor		1–10.7%	[58,59,60]
*AURKA*	Chromosome segregation, bipolar spindle microtubule formation, cytokinesis, mitosis exit	overexpression	Poor prognostic factor		51.6–72%	[61,62,63,64]

**Table 3:** Examples of lymphocyte subtypes infiltrating tumors and their association with prognosis.

Subtype	Molecular Determinant	Mechanism of Action	Prognosis	References
*Tc*	*CD3+ CD8+*	Cytotoxic killing of tumor cells Granzyne/perforin complex	Positive	[115,116]
*Th1*	*CD3+ CD4+*	Activation of *CD8+* T-cell mediated cell killing Production of *IFN-γ*, *IL-2*, *IFN-α*	Positive	[115,116]
*Th2*	*CD3+ CD4+*	Activation of humoral response Production of *IL-4*, *IL-5*	Positive	[115]
			Negative	[115,117,118]
Treg	*CD3+ CD4+ CD25+ Foxp3+*	Immunosuppression Induction of immune-tolerance	Negative	[119]
B-cells, Plasma cells	*CD19+* *CD20+* *CD38+*	Antibody-dependent cell death Presentation of tumor antigens to T-cells	Positive	[108,120]
		Production of inhibitory factors	Negative	[121]
NK	*CD56+* *CD3−*	Innate immune cytotoxicity	Positive	[116]

**Table 4:** Examples of the most common non-lymphocyte cell types infiltrating tumors and their association with prognosis.

Subtype	Molecular Determinant	Mechanism of Action	Prognosis	References
Tumor-associated macrophages (*TAMs*)—M1	*CD68+*	Inflammatory response Induction of Th1 response	Positive	[110,116]
Tumor-associated macrophages (*TAMs*)—M2	*CD163+*	Increasing proliferation Render poorer differentiation Promotion of angiogenesis Promotion of metastasis Secretion of IL-10 Inhibition of Th1 response	Negative	[13,116]
Tumor-Associated Neutrophils (*TANs*) N1	*CD66b+* *CD15-*	Direct lysis of tumor cells Induction of antitumor cytotoxicity	Positive	[116,122]
Tumor-Associated Neutrophils (*TANs*) N2	*CD66b+* *CD15+*	Promotion of tumor proliferation Promotion of tumor migration Promotion of tumor invasion and metastasis Inhibition of antitumor immunity	Negative	[116,123,124]
Cancer-associated fibroblasts (*CAFs*)	α-SMA	Reduction in antitumor immunity Enhancement of proliferation and invasion Promotion of neoangiogenesis Reshape the extracellular matrix Formation of an immunosuppressive microenvironment	Negative	[116]
Cancer-Associated Adipocytes (*CAAs*)	Depends on the type. e.g.: *UCP1*, *MYF5*, *EVA1*, *CD137*, *TBX1*	Secretion of *CCL2*, *CCL5*, *IL-1*, *IL-6*, *TNF-α*, *VEGF* Promote tumor cell proliferation and invasion Promote angiogenesis	Negative	[116,125]

**Table 5:** Clinical evidence of immunomodulators in immunotherapy of *TNBC*.

Immunomodulators	Study Details	Results	References
Atezolizumab	Atezolizumab intravenously (15 or 20 mg/kg, or at a 1200-mg flat dose), every 3 weeks in metastatic *TNBC* (required ≥5% PD-L1 positivity) (*n* = 116); NCT01375842	Efficacy, manageable safety profile, well tolerated, adverse events: fatigue, nausea, diarrhea, hypothyroidism, asthenia, decreased appetite, arthralgia, pruritus, or rush	[138,139]
Pembrolizumab	Pembrolizumab (200 mg) administered intravenously over 30 min every 3 weeks for up to 2 years in *TNBC* women (*n* = 170) with PD-L1-positive tumors (61.8%) and received ≥3 previous lines of therapy for metastatic disease (43.5%); NCT02447003	Manageable safety profile and durable antitumor activity, well-tolerated; common adverse events: fatigue, nausea, hypothyroidism, decreased appetite, diarrhea, asthenia, pruritus, arthralgia, or hyperthyroidism	[140]
	Intravenous pembrolizumab at 10 mg/kg every 2 weeks to *TNBC* patients with advanced PD-L1-positive (*n* = 111); NCT01848834	Clinical activity and potentially acceptable safety profile of pembrolizumab; mild toxicities: arthralgia, fatigue, myalgia, and nausea	[152]
	Intravenous pembrolizumab (200 mg once every 3 weeks for 35 cycles) (*n* = 312) or single-drug chemotherapy (capecitabine, eribulin, gemcitabine, or vinorelbine) (*n* = 310) in *TNBC* patients stratified according to PD-L1 positivity/negativity; NCT02555657	Not significant effect for improving overall survival; common adverse events: anaemia, decreased white blood cells or neutrophil count, and neutropenia	[153]
	Pembrolizumab (200 mg in first cycle), then eight cycles of pembrolizumab in combination with a taxane with or without carboplatin for 12 weeks, and then doxorubicin and cyclophosphamide for 12 weeks before surgery in patients with high-risk, early-stage *TNBC* (*n* = 60); NCT02622074	Toxicity and promising antitumor activity related to positive correlation with tumor PD-L1 expression and stromal tumor-infiltrating lymphocyte levels; common adverse events: neutropenia	[154]
Atezolizumab and nab-paclitaxel (FDA-approved combination for unresectable locally advanced or metastatic PD-L1 positive *TNBC*)	Intravenous atezolizumab (800 mg) on days 1 and 15 of each cycle every 2 weeks and intravenous nab-paclitaxel (125 mg/m^2^) on days 1, 8, and 15 of each cycle (3 weeks on, 1 week off) in women (*n* = 33) with stage IV or locally recurrent *TNBC* and 0 to 2 lines of prior chemotherapy in the metastatic setting; NCT01633970	Standard treatment, safe and clinically active; common adverse events: neutropenia, fatigue, alopecia, diarrhea, peripheral sensory neuropathy, peripheral neuropathy, and nausea	[141,142]
	Intravenous atezolizumab (840 mg) on day 1 and day 15 of every 28-day cycle and intravenous nab-paclitaxel (100 mg/m^2^ of body surface area) on days 1, 8, and 15 until progression or unacceptable toxicity in *TNBC* patients (*n* = 451) and placebo group (*n* = 451); NCT02425891	Higher overall survival in the patients treated with Atezolizumab and nab-paclitaxel (21·0 months) when compared with placebo group (18·7 months); common adverse events: neutropenia, peripheral neuropathy, decreased neutrophil count, and fatigue; Treatment-related deaths (*n* = 2) due to autoimmune hepatitis and septic shock	[155]
Atezolizumab and entinostat	Atezolizumab (1200 mg) + entinostat (5 mg) in patients with advanced *TNBC* (*n* = 81); NCT02708680	Not prolonged progression-free survival, greater toxicity of combination when compared to atezolizumab or placebo treated group	[156]
Niraparib combined with Pembrolizumab	Oral niraparib (200 mg of once daily) in combination with intravenous pembrolizumab (200 mg on day 1) of each 21-day cycle) in patients with advanced or metastatic *TNBC* (*n* = 55) irrespective of *BRCA* mutation status or *PD-L1* expression; NCT02657889	Promising antitumor activity, especially with higher response rates in patients with tumor BRCA mutations; common adverse events: anemia, thrombocytopenia, and fatigue	[157].
Pembrolizumab combined with radiotherapy	Pembrolizumab (200 mg) was given intravenously within 3 days of first radiotherapy, then every 3 weeks +/−3 days until disease progression in metastatic *TNBC* patients (*n* = 17); NCT02730130	Well-tolerated combination, stable disease (*n* = 1) and decreased tumor burden (*n* = 3); common adverse events: mild fatigue, myalgia, and nausea	[158]
Pembrolizumab combined with eribulin	Intravenous administration of pembrolizumab (200 mg on day 1 of 21-day cycles) with intravenous eribulin (1.4 mg/m^2^ on day 1 and day 8) in patients with metastatic *TNBC* (*n* = 160); NCT02513472	Well-tolerated combination with promising antitumor activity, higher objective response rate in patients with PD-L1-positive tumors; common adverse events: fatigue, nausea, peripheral sensory neuropathy, alopecia, and constipation	[159]
Pembrolizumab combined with Imprime PGG	Pembrolizumab (200 mg on D1 of each cycle) and Imprime (4 mg/kg IV days 1, 8, 15 of each 3-week cycle) in patients with metastatic *TNBC*, Simon 2 stage study (*n* = 12 Stage 1, *n* = 32 Stage 2); NCT02981303	Well-tolerated combination, innate immune activation through increased *CD86* on circulating monocytes and *CD8* T cell activation (*PD1+/Ki67+/HLA-DR+*)	[160]
